# Assistência farmacêutica em acesso expandido, uso compassivo e fornecimento de medicamento pós-estudo na perspectiva de pesquisadores clínicos

**DOI:** 10.1590/0102-311XPT128824

**Published:** 2025-06-27

**Authors:** Maria Bárbara Faria Cardoso da Silva, Cecilia Ferreira da Silva, Mario Jorge Sobreira da Silva

**Affiliations:** 1 Instituto Nacional de Câncer, Rio de Janeiro, Brasil.; 2 Escola Nacional de Saúde Pública Sergio Arouca, Fundação Oswaldo Cruz, Rio de Janeiro, Brasil.

**Keywords:** Assistência Farmacêutica, Programa de Acesso Expandido, Ensaio Clínico, Fase IV, Estudo de Caso, Pharmaceutical Services, Expanded Access Trial, Clinical Trial, Phase IV, Case Study, Servicios Farmacéuticos, Ensayos de Uso Compasivo, Ensayo Clínico Fase IV, Estudio de Caso

## Abstract

Este estudo busca compreender os fluxos da assistência farmacêutica e as contribuições do farmacêutico nos programas de acesso expandido, de uso compassivo e de fornecimento de medicamento pós-estudo, sob a perspectiva dos profissionais de saúde com experiência em pesquisa clínica. Realizou-se um estudo de caso em um hospital de oncologia do Rio de Janeiro, seguido de aplicação de questionário envolvendo farmacêuticos de diferentes regiões do Brasil. O estudo foi fundamentado no referencial teórico do interacionismo interpretativo de Denzin. Os dados foram analisados pela técnica de construção da explicação e interpretados de acordo com a análise temática de conteúdo. Identificou-se três eixos temáticos: operacionalização da assistência farmacêutica, atribuições clínicas do farmacêutico e melhoria contínua da qualidade. Participaram dez profissionais na etapa do estudo de caso e 12 responderam ao questionário. Os resultados indicaram necessidades convergentes, destacando a importância da otimização das atividades logísticas e operacionais, o acompanhamento farmacoterapêutico, a educação permanente, a farmacovigilância, a informação e a qualidade em saúde. Os dados gerados têm o potencial de orientar futuros documentos para aprimorar os processos relacionados aos programas, preenchendo lacunas da regulamentação e promovendo melhor suporte à assistência à saúde.

## Abstract (en)

This study sought to understand the flows of pharmaceutical services and the contributions of pharmacists in expanded access programs, compassionate use, and the post-trial access of medications, from the perspective of healthcare professionals experienced in clinical research. A organizacional case study was conducted at an oncology hospital in Rio de Janeiro, followed by the administration of a questionnaire to pharmacists from different regions of Brazil. The study was grounded in Denzin’s theoretical framework of interpretive interactionism. Data were analyzed using an explanatory construction technique and interpreted via thematic content analysis. In total, three thematic axes emerged: the operationalization of pharmaceutical services; the clinical responsibilities of the pharmacist; and continuous quality improvement. A total of ten professionals participated in the organizational case study phase, and 12 responded to the questionnaire. The results indicated convergent needs, underscoring the importance of optimizing logistical and operational activities, drug therapy monitoring, continuing education, pharmacovigilance, information dissemination, and healthcare quality. These findings have the potential to inform future guidelines to enhance program processes, address regulatory gaps, and provide better support for healthcare.

## Resumen

Este estudio buscó identificar los flujos de atención farmacéutica y las aportaciones del farmacéutico en los programas de acceso ampliado, uso compasivo y suministro de fármacos postestudio desde la perspectiva de profesionales de la salud con experiencia en investigación clínica. Se realizó un estudio de caso en un hospital oncológico de Río de Janeiro, seguido de la aplicación de un cuestionario que reunió a farmacéuticos de distintas regiones de Brasil. El estudio se basó en el marco teórico del interaccionismo interpretativo de Denzin. Para el análisis de los datos se aplicó la técnica de construcción de la explicación, y para interpretarlos se utilizó el análisis de contenido temático. Se identificaron tres ejes temáticos: operacionalización de la atención farmacéutica; funciones clínicas del farmacéutico; mejora continua de la calidad. Diez profesionales participaron en la etapa de estudio de caso y 12 respondieron el cuestionario. Los resultados indicaron necesidades convergentes y destacan la importancia de optimizar la logística y las actividades operativas, el seguimiento farmacoterapéutico, la educación continua, la farmacovigilancia, la información y la calidad en salud. Los datos generados tienen el potencial de servir de guía a los documentos futuros para mejorar los procesos relacionados con los programas, lo cual llena vacíos regulatorios y promueve un mejor apoyo para la atención médica.

## Introdução

O controle do câncer é realizado de forma multimodal e, para isso, os usuários do sistema de saúde devem dispor de tratamento oportuno, eficaz e, se possível, inovador. Parte do acesso à medicamentos inovadores pode ser proporcionado por meio da pesquisa clínica de qualidade e de programas de fornecimento de novas tecnologias oriundos desta primeira ^1^ ^,^ ^2^.

Os programas de acesso expandido (PAE), o uso compassivo (PUC) e o fornecimento de medicamento pós-estudo (FMPE) são regulamentados no Brasil e visam fornecer medicamento novo, promissor e ainda sem registro na Agência Nacional de Vigilância Sanitária (Anvisa). A anuência desses depende da gravidade e estágio da doença, da ausência de alternativa terapêutica satisfatória no país, da presença de comorbidades e da avaliação da relação risco-benefício do uso do medicamento ^3^.

As ações e serviços farmacêuticos envolvendo os produtos sob investigação - aqueles que se encontram em fase de estudo clínico ^4^ - e os medicamentos já aprovados para uso no país estão adequadamente definidos dentro do ciclo da assistência farmacêutica ^5^. Entretanto, no âmbito dos PUC, PAE e FMPE não há uma delimitação quanto às atividades que devem ser desempenhadas pelos profissionais de saúde. Tais programas são considerados, por muitos autores e normativas, como utilização de medicamentos inovadores nos moldes de “vida real”, sem o controle rígido de um estudo clínico - mas utilizando um produto sob investigação ^6^ ^,^ ^7^.

Os medicamentos fornecidos pelos programas a uma população vulnerável não possuem um fluxo padrão de assistência farmacêutica definido, nem descritos em literatura disponível na forma de diretrizes ou guias. Apenas a Espanha apresenta um consenso sobre procedimentos a serem adotados por farmacêuticos em casos de fornecimento de produtos sob investigação por intermédio de PUC, PAE e FMPE ^8^. No Brasil, a atuação do farmacêutico nesse contexto não possui um direcionamento ^9^, principalmente no contexto do Sistema Único de Saúde (SUS).

O objetivo deste estudo foi compreender as atividades da assistência farmacêutica e as contribuições do farmacêutico nos PUC, PAE e FMPE sob a perspectiva dos profissionais de saúde com experiência em pesquisa clínica.

## Métodos

Realizou-se um estudo de caso envolvendo a equipe multiprofissional da pesquisa clínica de um hospital referência em oncologia no Estado do Rio de Janeiro, Brasil, e a aplicação de um questionário eletrônico a farmacêuticos especialistas de diferentes regiões do país - envolvidos com os PUC, PAE e FMPE em sua rotina assistencial.

No estudo de caso foram incluídos profissionais de saúde atuantes diretamente com qualquer etapa da cadeia da assistência farmacêutica (planejamento, regulatório, qualidade, solicitação, recebimento, armazenamento e distribuição) relacionada aos medicamentos fornecidos pelos programas em onco-hematologia. Excluíram-se profissionais com menos de um ano de experiência na área. Na etapa de aplicação dos questionários, incluíram-se farmacêuticos possuindo no mínimo um ano de experiência com os programas. Utilizou-se a técnica de bola de neve para atingir o maior número possível de profissionais na amostra ^10^.

A coleta de dados do estudo de caso foi realizada pela aplicação de entrevistas presenciais ou por videoconferência após a obtenção do Termo de Consentimento Livre e Esclarecido (TCLE). Durante as entrevistas, os participantes tiveram seus dados pessoais codificados, impedindo sua identificação.

Foram realizadas entrevistas com base em um roteiro estruturado, contendo perguntas abertas e fechadas. Para fins de transcrição, o áudio das entrevistas foi gravado após autorização dos envolvidos. Coletaram-se as seguintes informações: profissão, local de atuação, tempo de experiência na profissão, tempo de experiência em pesquisa clínica, atividades desenvolvidas relacionadas aos programas, experiência com os programas e seu respectivo tempo, atuação profissional nos programas, fatores facilitadores para a atuação profissional, fatores limitantes para a atuação profissional, contribuições farmacêuticas para os programas.

De forma complementar, foi utilizado um roteiro para observação direta da rotina dos profissionais farmacêuticos da pesquisa clínica envolvidos no fornecimento dos medicamentos dos programas. Observações do cenário de trabalho e impressões obtidas, durante e após os encontros, foram registradas em um diário de campo pelo pesquisador. Ademais, foi realizada uma análise documental dos dossiês dos programas. Optou-se pelo uso de variadas fontes de evidências para ampliar a validade interna do estudo ^11^ ^,^ ^12^.

Na etapa seguinte, foi utilizado um questionário eletrônico incluindo as mesmas perguntas do roteiro de entrevista aplicado no estudo de caso, com a finalidade de garantir a validade externa dos resultados.

O material obtido nas duas etapas foi compilado, tabulado e armazenado em um banco de dados exclusivo do estudo. Os dados foram organizados e analisados qualitativamente com o auxílio do software QDA Miner Lite (Free Edition) v2.0.9 (https://provalisresearch.com).

O referencial analítico empregado no estudo foi baseado no interacionismo interpretativo proposto por Denzin ^13^. Segundo o autor, são estabelecidas conexões entre os conteúdos descritos a partir das experiências pessoais dos entrevistados, com as estruturas disponíveis e as políticas públicas vigentes. O resultado dessas análises proporciona um arcabouço denso de informações, tornando o material significativo ao envolver um conjunto de aspectos contextuais relevantes ^13^.

No interacionismo interpretativo, é esperado que, ao final das análises, sejam realizadas proposições de melhorias para os processos analisados baseadas nas vivências dos entrevistados e em mudanças necessárias aos programas, serviços ou atividades diante das perspectivas esperadas ^13^.

De forma operativa, os dados foram analisados pela técnica de construção da explicação ^14^. Utilizou-se a análise temática de conteúdo para analisar os resultados. Essa técnica permite que, a partir da descrição e interpretação do conteúdo da pesquisa - e após um processo de sistematização e categorização dos dados - seja possível obter um resultado válido e confiável ^11^.

Os dados organizados foram explorados, codificados e categorizados considerando o referencial teórico do ciclo da assistência farmacêutica ^5^ ^,^ ^15^. As subcategorias e os temas foram definidos *a posteriori*, conforme as características em comum identificadas nas entrevistas. Esse processo foi revisado e checado por outros dois pesquisadores. Ao final, emergiram três eixos temáticos: (a) Operacionalização da assistência farmacêutica; (b) Atribuições clínicas do farmacêutico; e (c) Melhoria contínua da qualidade.

O estudo atendeu às normativas ético-regulatórias, sendo aprovado pelo Comitê de Ética em Pesquisa do Instituto Nacional de Câncer (CEP-INCA; CAAE: 54563321.7.0000.5274).

## Resultados e discussão

No estudo de caso, as entrevistas foram realizadas com dez profissionais, sendo dois farmacêuticos, três enfermeiros, dois biomédicos, dois médicos e um biólogo. As entrevistas ocorreram entre abril e maio de 2022 e duraram, em média, 22,47 minutos, variando entre 11,32 e 37,12 minutos.

Os profissionais ocupavam os seguintes cargos na pesquisa clínica: dois farmacêuticos, dois pesquisadores, dois gestores, dois coordenadores de estudos clínicos, um analista regulatório e um analista de qualidade.

O tempo médio de experiência profissional foi de 12 anos, variando entre 3 e 21. Quanto à experiência em pesquisa clínica, apresentavam, em média, sete anos e quatro meses, com mínimo de um ano e seis meses e máximo de 17 anos. Em relação à experiência com os programas, sete tinham experiência com PUC, oito com PAE e nove com FMPE.

Em relação à atuação profissional, todos realizavam pelo menos uma das atividades da cadeia da assistência farmacêutica: oito na área logística (sendo oito com planejamento, quatro em gestão, três na garantia de qualidade e dois com recebimento, armazenamento e dispensação); quatro em áreas administrativas (sendo três em atividades regulatórias, um em contratos e um em gestão financeira); dois na área clínica com seguimento farmacoterapêutico; e três na área gerencial, realizando a orientação do processo como um todo.

O Quadro 1 apresenta as características dos profissionais entrevistados.

**Quadro 1:** Perfil dos profissionais da equipe multiprofissional participantes das entrevistas.

PROFISSIONAL ENTREVISTADO	TEMPO DE EXPERIÊNCIA NA PROFISSÃO	TEMPO DE EXPERIÊNCIA EM PESQUISA CLÍNICA	TEMPO DE EXPERIÊNCIA COM PUC	TEMPO DE EXPERIÊNCIA COM PAE	TEMPO DE EXPERIÊNCIA COM FMPE	ATIVIDADES DESENVOLVIDAS RELACIONADAS AOS PROGRAMAS
1	12 anos	8 anos	8 anos	8 anos	8 anos	Armazenamento; dispensação; acompanhamento farmacoterapêutico; planejamento; recebimento
2	18 anos	15 anos	7,5 anos	7,5 anos	7,5 anos	Qualidade; regulatório
3	12 anos	2 anos	Não	Não	1 ano	Regulatório
4	7 anos	4 anos	3 meses	7 meses	4 anos	Armazenamento; dispensação; acompanhamento farmacoterapêutico; planejamento; recebimento
5	3 anos	1,5 ano	Não	3 meses	1 ano	Planejamento
6	9 anos	7 anos	6 anos	6 anos	6 anos	Gestão; acompanhamento farmacoterapêutico; planejamento; qualidade; regulatório
7	16 anos	5 anos	8 anos	8 anos	Não	Acompanhamento farmacoterapêutico
8	4 anos	2 anos	Não	Não	1 ano	Gestão; planejamento; qualidade
9	18 anos	13 anos	13 anos	13 anos	13 anos	Gestão; acompanhamento farmacoterapêutico; planejamento
10	21 anos	17 anos	10 anos	10 anos	15 anos	Contrato; financeiro; gestão; planejamento

FMPE: fornecimento de medicamento pós-estudo; PAE: programa de acesso expandido; PUC: programa de uso compassivo.

Fonte: elaboração própria.

Nota: o termo “financeiro” está relacionado ao reembolso à instituição pela indústria farmacêutica (patrocinador) e/ou ao reembolso ao paciente.

Doze farmacêuticos especialistas de diferentes regiões do Brasil responderam ao questionário enviado eletronicamente, sendo um da Região Nordeste (Ceará), nove do Sudeste (Rio de Janeiro e São Paulo) e dois do Sul (Rio Grande do Sul). Destes, seis profissionais atuavam na rede privada e seis no setor público.

Quanto ao tempo de experiência como farmacêuticos, a maioria apresentava mais de oito anos. A experiência em pesquisa clínica variou entre um e mais de oito anos. Entre os 12 especialistas, 11 tinham experiência com PUC, oito com PAE e dez com FMPE. Alguns profissionais apresentavam experiência em apenas um dos programas porém possuíam conhecimento sobre os três.

**Figura 1:**
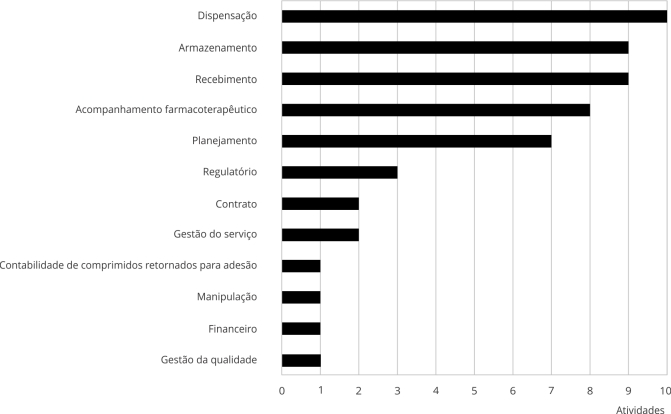
Atividades da assistência farmacêutica autorrelatadas pelos farmacêuticos respondentes ao questionário relacionadas aos programas de acesso expandido (PAE), uso compassivo (PUC) e fornecimento de medicamento pós-estudo (FMPE).

As atividades da assistência farmacêutica desenvolvidas pelos farmacêuticos especialistas, captadas pela resposta ao questionário, foram compiladas na Figura 1.

Fonte: elaboração própria. Nota: o termo “financeiro” está relacionado ao reembolso à instituição pela indústria farmacêutica (patrocinador) e/ou ao reembolso ao paciente.

### Quanto à “Operacionalização da assistência farmacêutica”

A assistência farmacêutica é estruturada por diversos elementos que compõem um ciclo de atividades inter-relacionadas, tornando-a operacional e servindo de modelo para desenhar ou melhorar os serviços de farmácia ^5^.

A análise da operacionalização da assistência farmacêutica no cenário do estudo trouxe as seguintes subcategorias, considerando as falas dos entrevistados: estrutura organizacional e processo de trabalho.

Identificou-se na estrutura organizacional uma complexidade ^16^ na solicitação dos programas pelos médicos responsáveis, bem como na execução das etapas da assistência farmacêutica conduzidas por diversos profissionais se comparado com estudos clínicos. Essa é uma característica inerente desses programas assistenciais ^3^ ^,^ ^9^, seja por seus aspectos regulatórios, seja por particularidades documentais, por exemplo. O exposto é evidenciado pelas falas dos entrevistados 9 e 7.

“*...o processo de solicitação do medicamento é extremamente trabalhoso... cadastramento no site, aí você tem que fazer a solicitação formal... depois disso você tem o acompanhamento do paciente... avaliação da decisão de interrupção da droga... tudo fora de um protocolo de pesquisa, mas você acaba tendo uma condução e função no dia a dia muito parecida...*” (entrevistado 9).

“*...entrar em contato com o patrocinador, solicitar, tem site, coisas que é a mão, mas tudo que está lá, que precisa de documentação, é a gente* [médicos] *que providencia. Desde a assinatura do paciente, TCLE, termos de que entendeu que aquilo é um uso compassionado* [por exemplo]*, preenchimento de dados, qual endereço da farmácia, qual nome da pessoa que vai receber... Até entrar no fluxo de vocês* [da farmácia]*, esse pontapé inicial burocrático é todo nosso*” (entrevistado 7).

De acordo com os entrevistados, é fundamental estruturar a condução dos programas baseado em documentos claros e de acesso público, visando ao aperfeiçoamento dos fluxos para melhorar o atendimento das necessidades dos usuários. As falas demonstraram que a forma de conduzir os programas é apreendida apenas com a prática, baseado na experiência dos profissionais. Tal cenário se configura pela inexistência de guias orientadores sobre os processos e devido às resoluções vigentes não subsidiarem a prática assistencial adequada ^16^ ^,^ ^17^.

Em contrapartida, houve relatos quanto à simplicidade dos processos relacionados aos programas se comparados aos ensaios clínicos:

“*Em questão de rotina, por não estar dentro daquelas obrigações que o estudo que está em andamento tem, rotina de monitoria, outras atividades, os programas são bem mais simples no andamento do estudo*” (entrevistado 4).

“*...o patrocinador só se compromete a entregar o medicamento, e aí a gente tem que fazer toda a parte né, vocês como farmacêuticos sabem que a gente não precisa passar aquele monte de dados, e toda aquela restrição que a gente tem no estudo clínico não é tão controlada, tanto no uso compassivo quanto no acesso expandido. Já é mais flexível*” (entrevistado 10).

As falas demonstram o ponto de vista da equipe, considerando ser mais simples conduzir os programas a partir da premissa que existem menos exigências regulatórias que os estudos clínicos. Essa característica pode ser considerada positiva, uma vez que o principal objetivo dos programas é garantir acesso a produtos sob investigação a pacientes que enfrentam doenças que ameaçam a vida sem opção de outro tratamento disponível ^3^. A legislação que os regulamenta corrobora com esse objetivo, já que os tipifica como programas assistenciais sem intenção de pesquisa. Logo, quanto mais facilitado o processo, maior a possibilidade de acesso ao tratamento ^16^ ^,^ ^17^.

A análise do processo de trabalho envolveu os temas da avaliação clínica, da solicitação, do armazenamento, da distribuição e da dispensação. Os relatos destacaram uma das lacunas que motivou este estudo: o acompanhamento clínico inadequado dos pacientes ^18^ - sinalizando para um dos pontos em que deveria haver maior atenção dos profissionais.

“*...no fornecimento pós-estudo, o paciente quando sai* [do estudo clínico]*, ele para de ser acompanhado pelos médicos da pesquisa e passa* [a ser acompanhado] *por médicos da instituição...* [A equipe da pesquisa] *é quem marca consulta, manda pedido de exame,* [o paciente] *está acostumado com todo esse padrão e depois de dois anos e meio volta para a rotina. Mas aí as coordenadoras ficam ajudando e tal, mas essa função não é obrigatória...*” (entrevistado 9).

“*Dessa maneira que se faz o acompanhamento* [clínico]*, porque com esses programas não acontece* [obrigatoriamente] *com imagem, por exemplo. Isso é feito como rotina da instituição. Então, isso eu vejo que fica furado, porque precisa ser reportado a cada três meses. Pode ser que essa informação se perca, até porque são estudos... que não estão com o coordenador* [de estudos clínicos] *direto, porque as vezes não é obrigatório. E mesmo quando é e tem coordenador, esse paciente vai ser mais atendido no hospital* [na rotina]*, e não pela pesquisa clínica. Então se o pesquisador não registra essa informação no prontuário, fica difícil*” (entrevistado 6).

A solicitação da participação nos PUC, PAE e FMPE se dá por contato do investigador médico com o patrocinador ou indústria farmacêutica e posterior pedido de autorização de realização para a Anvisa ^3^ ^,^ ^19^. Considerando o ciclo da assistência farmacêutica, a solicitação no caso dos programas, se equipara à programação e à aquisição do medicamento ^15^.

Cabe destacar que a solicitação de medicamento, no sentido de repor o estoque - na maioria das vezes -, é responsabilidade da equipe da farmácia, conforme destacou um dos entrevistados.

“*A farmácia também fica responsável pela parte de solicitação do medicamento, é algo que a gente pode perceber, que o paciente não vai ficar sem tratamento porque vai ter alguém que vai fazer essa solicitação*” (entrevistado 6).

A fala do entrevistado remete a uma das principais funções do farmacêutico - o controle de estoque. Esta tarefa é essencial para a adequada manutenção do tratamento do paciente mediante a solicitação ^20^. As atividades se entrelaçam justamente por estarem dentro de um processo de trabalho que é contínuo, conforme à lógica do ciclo da assistência farmacêutica ^5^.

Os farmacêuticos entrevistados citaram sobre suas principais funções quanto aos programas: armazenamento adequado com controle de temperatura e acompanhamento de estoque com devolutiva para o patrocinador, garantindo a solicitação - para a entrega no tempo correto - antes do retorno do paciente para nova dispensação.

Estes afirmaram, também, que o gerenciamento de estoque relacionado aos programas demanda maior atenção uma vez que não há um ressuprimento automático via sistema do patrocinador, como ocorreria nos estudos clínicos ^21^.

Quanto à atividade de dispensação, que é um ato privativo do farmacêutico ^20^, ela foi considerada pelo entrevistado 9 como sendo “*menos supervisionada*” quando comparada àqueles fornecidos pelos estudos clínicos que possuem um representante da indústria farmacêutica denominado monitor - que periodicamente verifica a correta condução do tratamento do paciente ^4^.

A fala desse entrevistado, porém, pode ser considerada uma visão limitada do processo, um desconhecimento do serviço prestado pelos farmacêuticos da instituição ^22^, ou um não entendimento do ato da dispensação em si.

Entretanto, deve-se ponderar que o profissional apontou essa falta de supervisão apenas se comparado a um estudo clínico, sem fazer juízo de valor do serviço prestado. Sendo assim, demonstra-se a importância de disseminação para toda a equipe de saúde sobre a relevância do profissional farmacêutico nos processos do ciclo da assistência farmacêutica, em especial na etapa de dispensação ^23^.

### Quanto às “Atribuições clínicas do farmacêutico”

Segundo a *Resolução nº 585/2013* do Conselho Federal de Farmácia (CFF) ^24^, as atribuições clínicas do farmacêutico são aquelas que “*constituem os direitos e responsabilidades desse profissional no que concerne a sua área de atuação*”. A Resolução destaca grupos de atribuições clínicas relativas ao cuidado com a saúde nos âmbitos individual e coletivo, à comunicação e educação em saúde, à gestão da prática, produção e aplicação do conhecimento, e elenca inúmeras atividades pertinentes ao profissional farmacêutico.

A subcategoria “informação em saúde” pode ser considerada um complexo de transmissão e/ou recepção de eventos relacionados ao cuidado em saúde. Sendo esta capaz de subsidiar a população em geral e gestores da área saúde, dentre outros, sobre: serviços prestados; materiais e medicamentos consumidos; força de trabalho envolvida; planejamento, controle e avaliação de ações e serviços de saúde ^25^.

As informações sobre os programas foram demandas de alguns entrevistados com o intuito de entender como ocorrem os PUC, PAE e FMPE, uma vez que é um processo de fornecimento de medicamento fragmentado e que depende de muitos profissionais para que aconteça.

“*Eu gostaria sim de ter a noção do todo, de quantos* [estudos] *nós temos no momento, de poder contribuir porque a qualidade, ela cuida dessa questão de evitar que a gente tenha erros, talvez reuniões mensais, trimestrais, passar um feedback de como estão os programas do centro, exemplificando o número de dispensações que tiveram em cada mês...*” (entrevistado 2).

“*...esses estudos ficam um pouco soltos, o status deles para a equipe como um todo, talvez seja legal ter uma reunião com a farmácia em relação a esses estudos. Quem tem a informação maior sobre o que está acontecendo, quem são os pacientes são vocês, então talvez uma comunicação mais ativa da farmácia com a equipe da pesquisa clínica como um todo. Talvez fosse legal pra gente entender, acho que só com isso a gente consegue tirar nossas dúvidas do dia a dia sobre o que seriam esses programas...*” (entrevistado 3).

Considerando os relatos, fica evidente o interesse e a necessidade da equipe em ter informações sobre os programas relacionadas tanto aos dados numéricos de produção assistencial quanto ao número de medicamentos dispensados e pacientes atendidos, por exemplo - além da possibilidade de elaboração de indicadores do serviço com esses dados compilados, podendo fomentar ações de gestão e de avaliação quanto à política pública relacionada aos programas ^26^. Cabe à farmácia e aos farmacêuticos fornecer esse tipo de dado, considerando que são os responsáveis pelo gerenciamento dos medicamentos nesses programas na instituição, conforme recomendações do CFF ^23^ ^,^ ^24^.

A farmacovigilância também foi um tema trazido pelos entrevistados considerando a responsabilidade inerente do farmacêutico sobre a dispensação do medicamento, aproximando-o do paciente e facilitando o controle dos eventos adversos.

“*...quantificar e graduar os eventos adversos que ocorreram...*” (entrevistado 2).

“*...eu acredito que vocês* [farmácia] *poderiam estar mais ligadas em relação aos eventos adversos, já que vocês têm o controle de dispensação...*” (entrevistado 6).

“*...acompanhamento de efeitos colaterais, isso tudo é bem-vindo da farmácia...*” (entrevistado 9).

Toklu & Mensah ^27^ reforçam que os farmacêuticos devem atuar de forma mais eficaz no sistema de notificação de reações adversas a medicamentos. Os autores afirmam que os órgãos reguladores deveriam fazer normativas para incentivar estes profissionais a se envolverem ativamente neste processo.

O entrevistado 6 ressaltou quanto à característica específica dos programas relacionada à aprovação como medicamento pela Anvisa, pois suscitam maior importância no monitoramento das reações adversas se comparado com medicamentos já aprovados pelo órgão regulador.

“*E é importante ressaltar, principalmente em PUC e PAE, é que são medicamentos que ainda não tem aprovação, então esse tipo de informação é muito importante para saber o que está influenciando diretamente no paciente, se realmente está sendo positivo*” (entrevistado 6).

A *RDC nº 406/2020* da Anvisa ^28^ reforça que, mesmo aprovados para comercialização, os medicamentos ainda precisam ter a relação risco-benefício avaliadas periodicamente, promovendo uma terapêutica segura e efetiva.

O cuidado farmacêutico pode ser considerado uma importante contribuição do farmacêutico para o cuidado individual com o intuito de otimizar o uso dos medicamentos e melhorar os resultados em saúde ^29^ ^,^ ^30^. A orientação aos pacientes faz parte das atribuições clínicas do farmacêutico relativas ao cuidado à saúde - nos âmbitos individual e coletivo - com o sentido de instruir e assistir a todos que prescrevem, distribuem e utilizam os medicamentos quanto ao uso correto ^24^. Esta atividade foi atribuída principalmente ao farmacêutico pelos entrevistados.

“*...é crucial assim essa orientação que vocês dão sobre como deve tomar, como deve armazenar em casa porque tem alguns pacientes que não tem esse tipo de orientação, que toma os medicamentos de forma errada, às vezes até esquece e é o tipo de orientação que a gente da coordenação* [de estudos clínicos] *não consegue dar, não pode dar*” (entrevistado 5).

“*...a própria orientação de farmácia ambulatorial, sem contar que é um aprendizado para a própria equipe porque é um medicamento super novo, que nem está aprovado no país ainda. Então, esse controle todo aí, orientações, isso tudo é bem vindo da farmácia...*” (entrevistado 9).

Ressalta-se a importância da orientação farmacêutica no cenário dos medicamentos inovadores, uma vez que estes não possuem todos os dados de segurança descritos ^28^. O exemplificado pelos relatos é corroborado por alguns autores como Holle et al. ^31^, que citam que uma das responsabilidades do farmacêutico inclui educar pacientes e cuidadores sobre suas terapias.

O acompanhamento farmacoterapêutico é definido como um serviço farmacêutico provido durante vários encontros com o paciente ^30^. O entendimento dos profissionais entrevistados sobre o que seria o acompanhamento variou: enquanto uns relataram que o acompanhamento influenciaria na segurança do tratamento, outros consideraram como um processo de monitoramento. A compreensão de que o acompanhamento está relacionado à verificação da adesão do paciente ao tratamento foi citada por dois profissionais (entrevistados 5 e 6).

A análise das falas demonstra algum conhecimento sobre o papel do farmacêutico no processo de cuidado em saúde - no intuito de contribuir com a atenção em saúde dada aos pacientes atendidos pelos programas. Entretanto, é possível identificar uma visão limitada da equipe sobre as possibilidades de atividades que o farmacêutico pode e deve exercer em benefício do paciente e da terapia utilizada. Atividades como conciliação de medicamentos, revisão da farmacoterapia e auxílio no manejo de problemas de saúde autolimitados não foram citadas e são importantes serviços farmacêuticos que podem ser implementados ^30^.

### Quanto à “Melhoria contínua da qualidade”

Esta categoria pode ser considerada como o empenho em conjunto e contínuo de profissionais de saúde, pacientes, cuidadores, cientistas, instituições públicas e privadas, líderes e professores em realizar mudanças que irão gerar resultados melhores para os usuários do serviço de saúde, para o desempenho aprimorado do sistema assistencial e para o maior progresso profissional ^32^. Diante desta perspectiva, os seguintes temas se destacaram nas entrevistas: educação permanente e qualidade em saúde.

A educação permanente em saúde objetiva educar os profissionais quanto a suas práticas ^33^ e foi uma necessidade reportada pelos profissionais. Um exemplo foi o relato do entrevistado 3, que explicitou seu desconhecimento em relação aos programas e que “*acharia legal um fluxo de educação continuada*” para os profissionais, demandando “*uma interação maior da farmácia com a equipe do centro em relação a esses programas específicos*”.

Importante ressaltar que há uma diferença entre educação continuada e educação permanente. A educação continuada é um meio de obtenção de conhecimento técnico-científico de forma sequenciada e cumulativa pelo profissional, por intermédio da escolarização formal, de aprendizados, de experimentações laborais e de envolvimento no âmbito corporativo ou social ^34^. A forma como foi citado pelo profissional indicava uma educação permanente justificando o uso do termo diferente do que o entrevistado citou.

O entrevistado 4 relatou que gostaria de contribuir com a orientação aos outros membros da equipe sobre os programas.

“*Orientação da disponibilidade dos programas ao corpo clínico, prescritores de forma indireta. Não é realizado diretamente como uma rotina. De forma mais direta com outros farmacêuticos do hospital, com as farmacêuticas que trabalham na parte da conciliação ou acompanhamento. Essas farmacêuticas geralmente sempre perguntam quais tipos de protocolos estão abertos*” (entrevistado 4).

O farmacêutico, na função de disseminador de informações, no âmbito da pesquisa clínica, tem importante papel na atualização da equipe de saúde com o uso de novas terapias ^31^ ^,^ ^35^.

O interesse em implementar ou modificar os cuidados em saúde por meio da construção de conhecimento - a partir de iniciativa ou ação adaptativa, revisão e reflexão - pode ser considerado o meio de se realizar a melhoria contínua da qualidade e a qualidade em saúde em si ^32^.

Nessa lógica, se destacou a necessidade de fluxos de trabalhos descritos. A demanda de fluxos melhor definidos foi identificada nas falas dos profissionais entrevistados. O entrevistado 1 destacou a importância da padronização dos formulários de solicitação do medicamento como forma de repor o estoque. Considerando que cada patrocinador vai ter um modelo, o que pode ser feito são treinamentos periódicos da equipe quanto às possíveis formas de solicitação de medicamento que são conhecidas na instituição do estudo.

“*Além da padronização que não existe, de um fluxo melhor definido; registro padronizado sobre continuação e descontinuação de tratamento... Fluxograma prático a nível local, institucional, a nível nacional também, é interessante que ele saiba como reagir estando em qualquer lugar do Brasil com esse paciente*” (entrevistado 6).

Quanto à análise dos dados pertinentes aos farmacêuticos especialistas, as atividades que apresentaram destaque foram as ações do componente da macrogestão da assistência farmacêutica relacionadas aos medicamentos fornecidos pelos programas. Estas são responsáveis por garantir o fornecimento adequado do tratamento relacionado à assistência direta ao paciente e estão em acordo com a literatura ^5^.

O emprego das perguntas abertas permitiu que os profissionais pudessem se expressar livremente, minimizando potenciais vieses do pesquisador ao elaborar as perguntas do questionário ^36^.

Os especialistas descreveram facilidades e pontos positivos - descritos no Quadro 2 - que corroboraram com as falas dos entrevistados no estudo de caso, e reforçaram a importância e necessidade da realização deste estudo. Um farmacêutico afirmou que o tema apresentado não é do conhecimento de muitas pessoas e elogiou a abordagem do estudo.

**Quadro 2:** Facilidades na execução das atividades dos programas e pontos positivos da existência destes descritos pelos farmacêuticos respondentes ao questionário.

FACILIDADES NA EXECUÇÃO DOS PROGRAMAS	PONTOS POSITIVOS QUANTO A EXISTÊNCIA DOS PROGRAMAS
1. Solicitação de reposição de estoque simples em comparação a um estudo clínico	1. Acesso a medicamentos inovadores
2. Menor burocracia com processos facilitados	2. Adequado suporte das indústrias farmacêuticas
3. Pacientes inseridos na rotina da instituição em comparação a um estudo clínico	3. Possibilidade de demonstrar segurança e eficácia dos medicamentos na população brasileira

Fonte: elaboração própria.

Os processos simplificados e facilitados coadunam com o propósito de possibilitar o acesso aos produtos sob investigação a pacientes sem opção terapêutica. Além de ter sido suscitada a importância da avaliação de segurança e eficácia na população brasileira, contribuindo para aumentar o volume de dados que podem ser utilizados na análise periódica do risco-benefício, considerando o perfil da nossa população ^16^ ^,^ ^22^ ^,^ ^28^. O suporte das indústrias farmacêuticas é descrito por Bunnik et al. ^16^, que têm criado plataformas online facilitando o processo de fornecimento dos medicamentos.

Os profissionais especialistas descreveram também limitações, como apresentado no Quadro 3.

**Quadro 3:** Limitações apontadas pelos farmacêuticos especialistas respondentes ao questionário.

LIMITAÇÕES DESCRITAS NO QUESTIONÁRIO
Periodicidade de visitas ao estabelecimento de saúde aumentado
Maior quantidade de medicamento oral dispensado
Aumento da possibilidade de uso inadequado
Acompanhamento clínico com exames de imagem prejudicado ou ausente
Solicitação manual *vs.* solicitação automática em comparação aos estudos clínicos
Falta de conhecimento ou de informação sobre os programas
Fluxos não definidos
Falta de padronização no processo de solicitação

Fonte: elaboração própria.

A maioria das limitações apresentadas pelos farmacêuticos especialistas haviam sido previamente elencadas pelos profissionais que participaram do estudo de caso, reafirmando a necessidade de um trabalho para delimitar e entender as dificuldades apontadas. Destaca-se que os achados similares, utilizando duas fontes de informação distintas, aumentam e garantem a validade do construto ^12^.

A interpretação dos apontamentos quanto ao acompanhamento dos pacientes em uso dos medicamentos não aprovados - fornecidos pelos programas - pode sugerir que estes têm o seguimento do tratamento pela rotina das instituições, como se fosse um tratamento aprovado pela Anvisa ^37^. Isto acarreta uma maior periodicidade de visitas ao consultório com consequente dispensação de medicamento oral aumentada para atender ao período sem retornar à instituição. Essas características foram consideradas prejudiciais ao paciente por possibilitar o uso inadequado desses medicamentos pela lacuna temporal do acompanhamento profissional - além do inadequado acompanhamento clínico com exames periódicos de imagem por não ser exigido pelos programas, exceto se a instituição de saúde o realiza por conduta interna. O longo tempo de retorno à instituição e a precariedade do acompanhamento podem ser atribuídos à sobrecarga do sistema de saúde, principalmente no SUS ^38^.

A dificuldade do controle e a solicitação de reposição do estoque - se comparado aos estudos clínicos, a falta de padronização e de fluxos definidos - foram descritas pelos farmacêuticos, bem como pelos profissionais entrevistados no estudo caso, sendo percepções generalizadas. Os profissionais da pesquisa clínica, em geral, comparam a sistematização dos estudos clínicos em fornecer medicamentos inovadores com o intuito de gerar dados baseados em evidência com os medicamentos inovadores fornecidos pelos programas ^4^ ^,^ ^39^. Almejam, assim, que essas regras que garantem a qualidade e segurança sejam seguidas nos PUC, PAE e FMPE.

A falta de disseminação de informação sobre a existência e a disponibilidade dos programas também foi considerada na concepção desse trabalho, como apresentada nas entrevistas. A difusão desse conhecimento foi atribuída ao profissional farmacêutico e faz jus às suas competências como profissional do medicamento ^24^.

As sugestões de contribuição, como farmacêuticos, em relação aos programas foram dispostas no Quadro 4.

**Quadro 4:** Sugestões de contribuições descritas pelos farmacêuticos respondentes ao questionário para os programas.

SUGESTÕES DE CONTRIBUIÇÃO DESCRITAS NA RESPOSTA AO QUESTIONÁRIO
Otimização logística do ciclo da assistência farmacêutica
Acompanhamento farmacoterapêutico
Educação permanente com outros profissionais da instituição envolvida
Farmacovigilância

Fonte: elaboração própria.

A otimização da logística da assistência farmacêutica envolvendo os medicamentos fornecidos pelos programas foi unânime. Um adequado e definido ciclo garante que o medicamento seja fornecido ao paciente de forma segura e eficiente. O mesmo ocorre com o acompanhamento farmacoterapêutico ainda que essa atividade não seja necessariamente determinada pelo patrocinador - como nos estudos clínicos - a fim de garantir um cuidado de excelência ^4^ ^,^ ^15^ ^,^ ^30^.

A educação permanente de outros profissionais e a farmacovigilância são atividades bem estabelecidas como sendo de qualificação do farmacêutico e descritas nas legislações vigentes ^23^ ^,^ ^24^.

As contribuições da equipe multiprofissional no estudo de caso e dos farmacêuticos especialistas foram essenciais. Identificou-se diversos pontos de oportunidades de melhoria. É importante destacar que o objetivo não era preencher todas as lacunas do tema, tão pouco apresentar as resoluções de todas as questões. A finalidade principal era detectar as necessidades do serviço apresentadas pelos profissionais que o executam, podendo fornecer subsídios para a gestão no processo de tomada de decisão. Nessa lógica, o estudo parece ter cumprido seu papel ao trazer uma visão realista das necessidades da área.

Uma limitação da pesquisa diz respeito ao número de participantes na etapa do estudo de caso, uma vez que nem todos foram entrevistados. Entretanto, percebeu-se a saturação dos dados durante as entrevistas realizadas. Ademais, o uso de outras fontes de informação, como a observação direta e a análise documental, parecem ter minimizado essa limitação potencial, uma vez que agregaram informações importantes para interpretação dos achados e permitiu obter um olhar abrangente sobre o fenômeno investigado. Ressalta-se, também, que a aplicação do questionário com os farmacêuticos especialistas coadunou os achados no estudo de caso, ampliando a sua validade.

## Considerações finais

Evidenciou-se no estudo que a falta de documentos oficiais é um fator que afeta a prestação de assistência à saúde segura e de qualidade relacionada aos PUC, PAE e FMPE. Os dados gerados na presente investigação poderão ser úteis para nortear a elaboração de um documento que oriente os processos relacionados aos programas e que, no futuro, poderá suprir uma lacuna existente, promovendo um benefício à saúde pública.

## References

[1] Silva CF, Lima LD, Osorio-de-Castro CGS (2021). Government funding of cancer research in Brazil. J Cancer Policy.

[2] Wild CP, Weiderpass E, Stewart BW (2020). World cancer report: cancer research for cancer prevention.

[3] Ministério da Saúde; Agência Nacional de Vigilância Sanitária (2013). Resolução da Diretoria Colegiada nº 38, de 12 de agosto de 2013. Aprova o regulamento para os programas de acesso expandido, uso compassivo e fornecimento de medicamento pós-estudo.. Diário Oficial da União.

[4] International Council for Harmonisation Integrated addendum to ICH E6(R1): guideline for good clinical practice E6(R2)..

[5] Silva MJS, Osorio-de-Castro CGS (2019). Organização e práticas da assistência farmacêutica em oncologia no âmbito do Sistema Único de Saúde. Interface (Botucatu).

[6] Dainesi SM, Goldbaum M (2011). Fornecimento de medicamento investigacional após o fim da pesquisa clínica - revisão da literatura e das diretrizes nacionais e internacionais. Rev Assoc Méd Bras.

[7] Miller JE, Ross JS, Moch KI, Caplan AL (2017). Characterizing expanded access and compassionate use programs for experimental drugs. BMC Res Notes.

[8] Busto F (2002). Documento de consenso para la prevención para la utilización de medicamentos en la modalidad de uso compasivo en la terapéutica onco/hematológica.

[9] Stout J, Smith C, Buckner J, Adjei AA, Wentworth M, Tilburt JC (2021). Oncologists' reflections on patient rights and access to compassionate use drugs a qualitative interview study from an academic cancer center. PLoS One.

[10] Biernacki P, Waldorf D (1981). Snowball sampling problems and techniques of chain referral sampling. Sociol Methods Res.

[11] Bardin L (2016). Análise de conteúdo..

[12] Silva MJS (2010). Avaliação da Farmácia Hospitalar em hospitais estaduais do Rio de Janeiro.

[13] Denzin NK (2001). Interpretive interactionism.

[14] Yin RK (2015). Estudo de caso: planejamento e métodos. 5ª Ed.

[15] Correr CJ, Otuki MF, Soler O (2011). Assistência farmacêutica integrada ao processo de cuidado em saúde gestão clínica do medicamento. Rev Pan-Amazônica Saúde.

[16] Bunnik EM, Aarts N, van de Vathorst S (2018). Little to lose and no other options ethical issues in efforts to facilitate expanded access to investigational drugs. Health Policy.

[17] Lewis K (2018). Post-trial access to treatment How managed access programs could be a solution. Med Access Point Care.

[18] Grover P, Babar ZUD, Oehmen R, Vitry A (2018). Medicines access programs to cancer medicines in Australia and New Zealand an exploratory study. Health Policy.

[19] Agência Nacional de Vigilância Sanitária (2019). Resolução da Diretoria Colegiada nº 311, de 10 de outubro de 2019. Altera a Resolução da Diretoria Colegiada - RDC nº 38, de 12 de agosto de 2013, que aprova o regulamento para os programas de acesso expandido, uso compassivo e fornecimento de medicamento pós-estudo.. Diário Oficial da União.

[20] Conselho Federal de Farmácia (2012). Resolução nº 568, de 6 de dezembro de 2012. Dá nova redação aos artigos 1º ao 6º da Resolução/CFF nº 492 de 26 de novembro de 2008, que regulamenta o exercício profissional nos serviços de atendimento pré-hospitalar, na farmácia hospitalar e em outros serviços de saúde, de natureza pública ou privada.. Diário Oficial da União.

[21] Ruikar V (2016). Interactive voice/web response system in clinical research. Perspect Clin Res.

[22] Barberato LC, Scherer MDA, Lacourt RMC (2019). O farmacêutico na atenção primária no Brasil uma inserção em construção. Ciênc Saúde Colet.

[23] Conselho Federal de Farmácia (2001). Resolução nº 357, de 20 de abril de 2001. Aprova o regulamento técnico das Boas Práticas de Farmácia.. Diário Oficial da União.

[24] Conselho Federal de Farmácia (2013). Resolução nº 585, de 29 de agosto de 2013. Regulamenta as atribuições clínicas do farmacêutico e dá outras providências.. Diário Oficial da União.

[25] Moreno AB, Coeli CM, Munck S Informação em saúde..

[26] Mosegui GBG, Antoñanzas F (2019). Normatização de programas de acesso expandido e uso compassivo de medicamentos na América do Sul. Rev Panam Salud Pública.

[27] Toklu HZ, Mensah E (2016). Why do we need pharmacists in pharmacovigilance systems. Online J Public Health Inform.

[28] Agência Nacional de Vigilância Sanitária (2020). Resolução da Diretoria Colegiada nº 406, de 22 de julho de 2020. Dispõe sobre as Boas Práticas de Farmacovigilância para Detentores de Registro de Medicamento de uso humano, e dá outras providências.. Diário Oficial da União.

[29] Allemann SS, van Mil JWF, Botermann L, Berger K, Griese N, Hersberger KE (2014). Pharmaceutical care the PCNE definition 2013. Int J Clin Pharmacy.

[30] Conselho Federal de Farmácia (2016). Serviços farmacêuticos diretamente destinados ao paciente, à família e à comunidade: contextualização e arcabouço conceitual.

[31] Holle LM, Segal EM, Jeffers KD (2020). The expanding role of the oncology pharmacist. Pharmacy (Basel).

[32] Batalden PB, Davidoff F (2007). What is "quality improvement" and how can it transform healthcare. Qual Saf Health Care.

[33] Ceccim RB, Ferla AA Educação permanente em saúde..

[34] Secretaria de Gestão do Trabalho e da Educação na Saúde.Ministério da Saúde (2009). Glossário temático: gestão do trabalho e da educação na saúde.

[35] Vulaj V, Hough S, Bedard L, Farris K, Mackler E (2018). Oncology pharmacist opportunities closing the gap in quality care. J Oncol Practice.

[36] Marques JBV, Freitas D (2018). Método DELPHI caracterização e potencialidades na pesquisa em Educação. Pro-Posições.

[37] Aquino T, Martinez L (2023). Programas assistenciais para medicamentos no Brasil nos últimos três anos (2019-2021) uso compassivo, acesso expandido e fornecimento de medicamento pós-estudo. Arq Méd Hosp Fac Ciênc Méd Santa Casa São Paulo.

[38] Souza PRB, Szwarcwald CL, Damacena GN, Stopa SR, Vieira MLFP, Almeida WS (2021). Cobertura de plano de saúde no Brasil análise dos dados da Pesquisa Nacional de Saúde 2013 e 2019. Ciênc Saúde Colet.

[39] Agência Nacional de Vigilância Sanitária (2015). Resolução da Diretoria Colegiada nº 9, de 20 de fevereiro de 2015. Dispõe sobre o Regulamento para a realização de ensaios clínicos com medicamentos no Brasil.. Diário Oficial da União.

